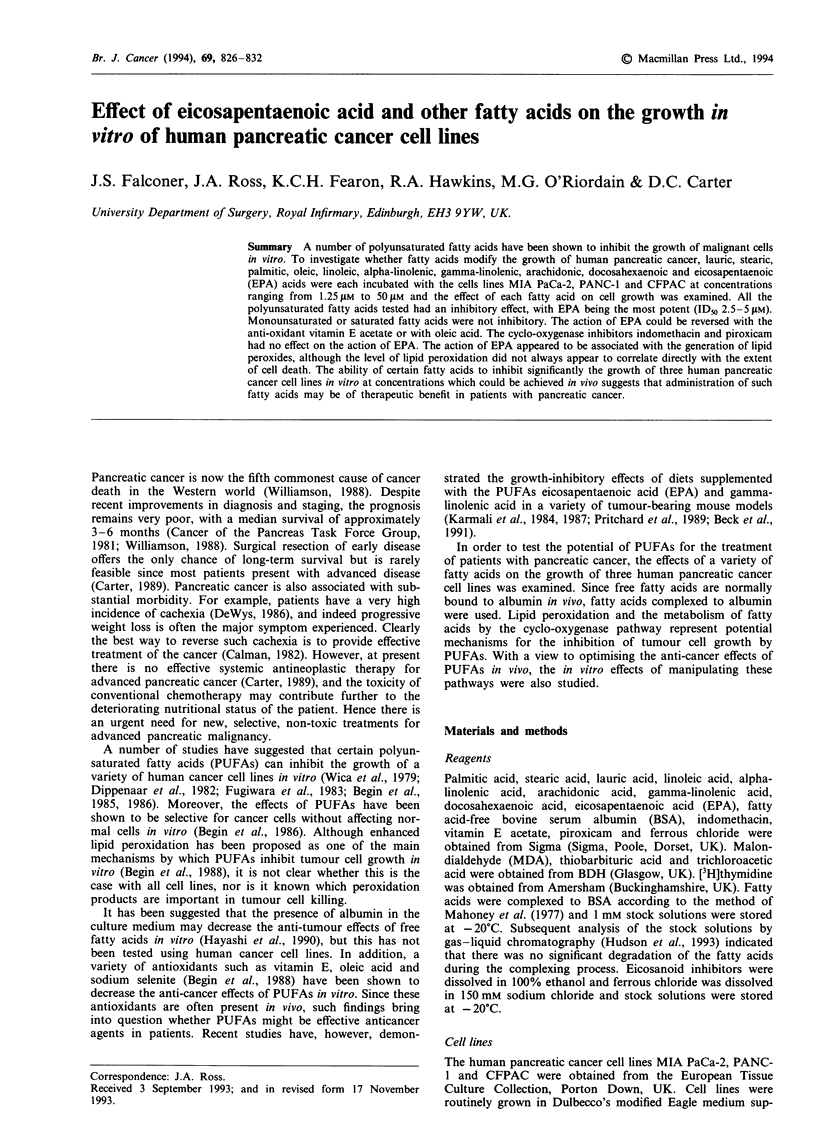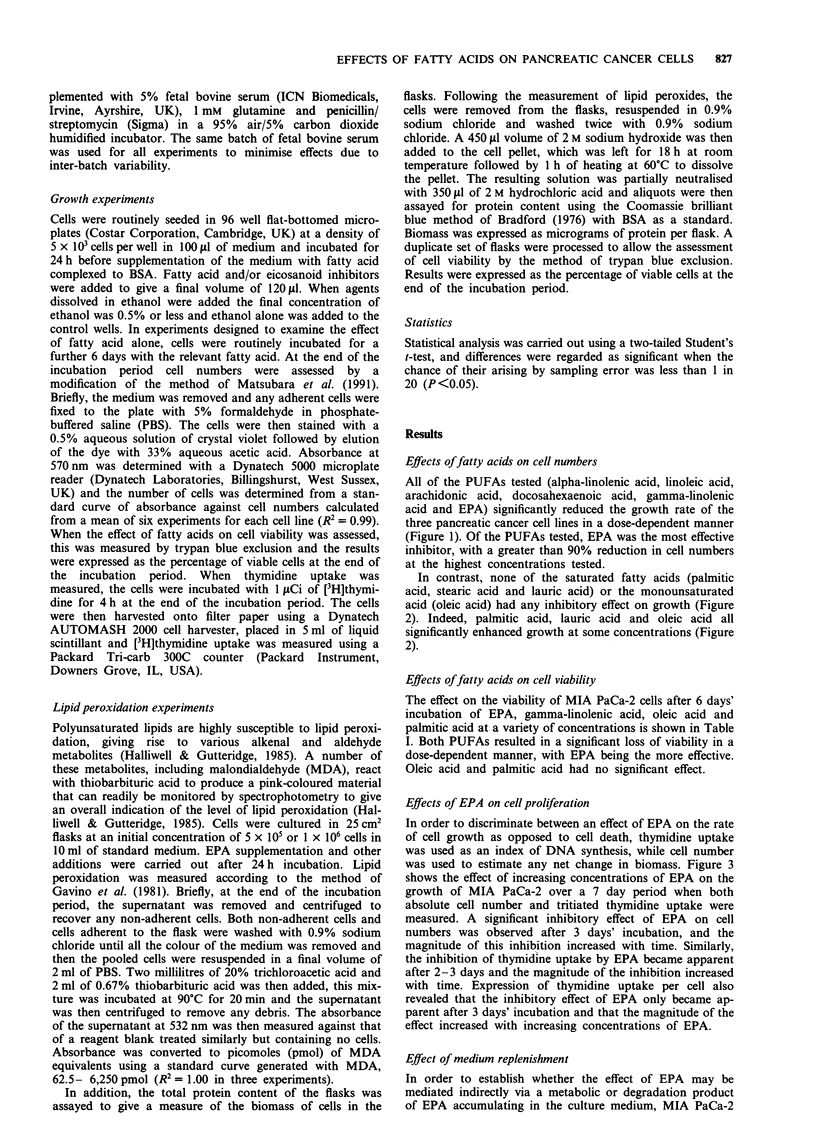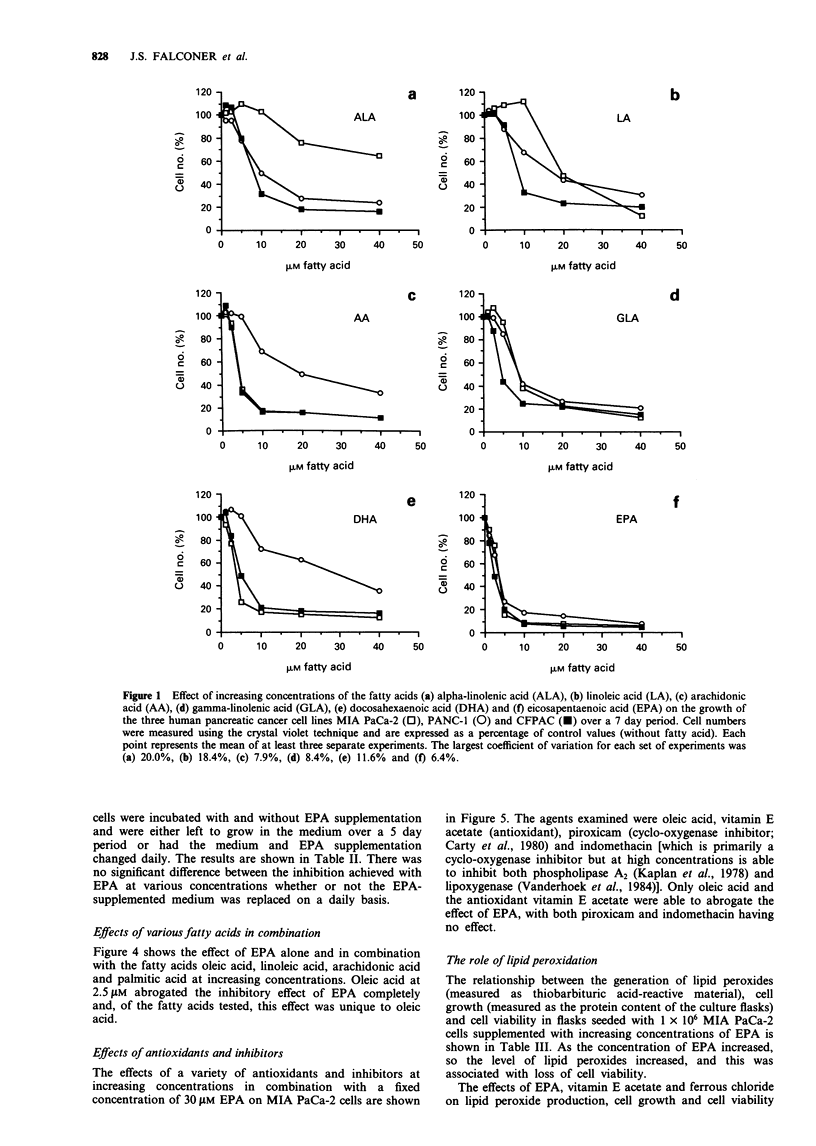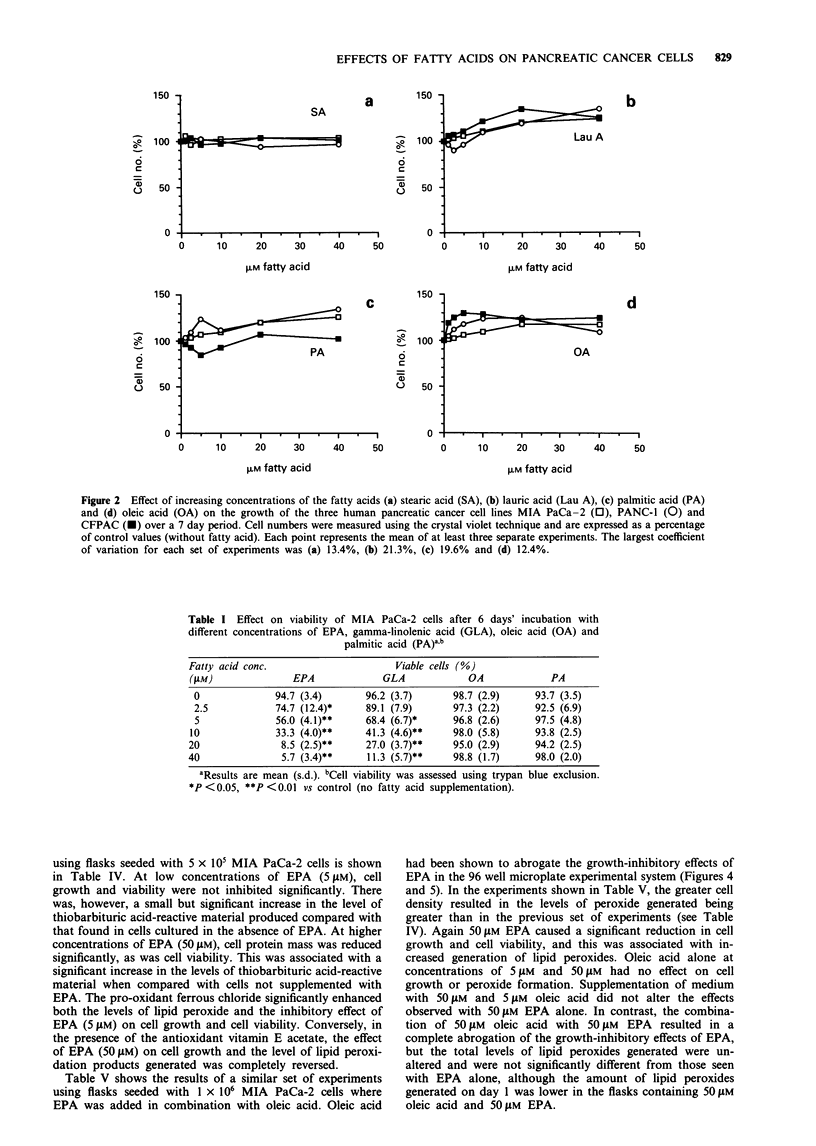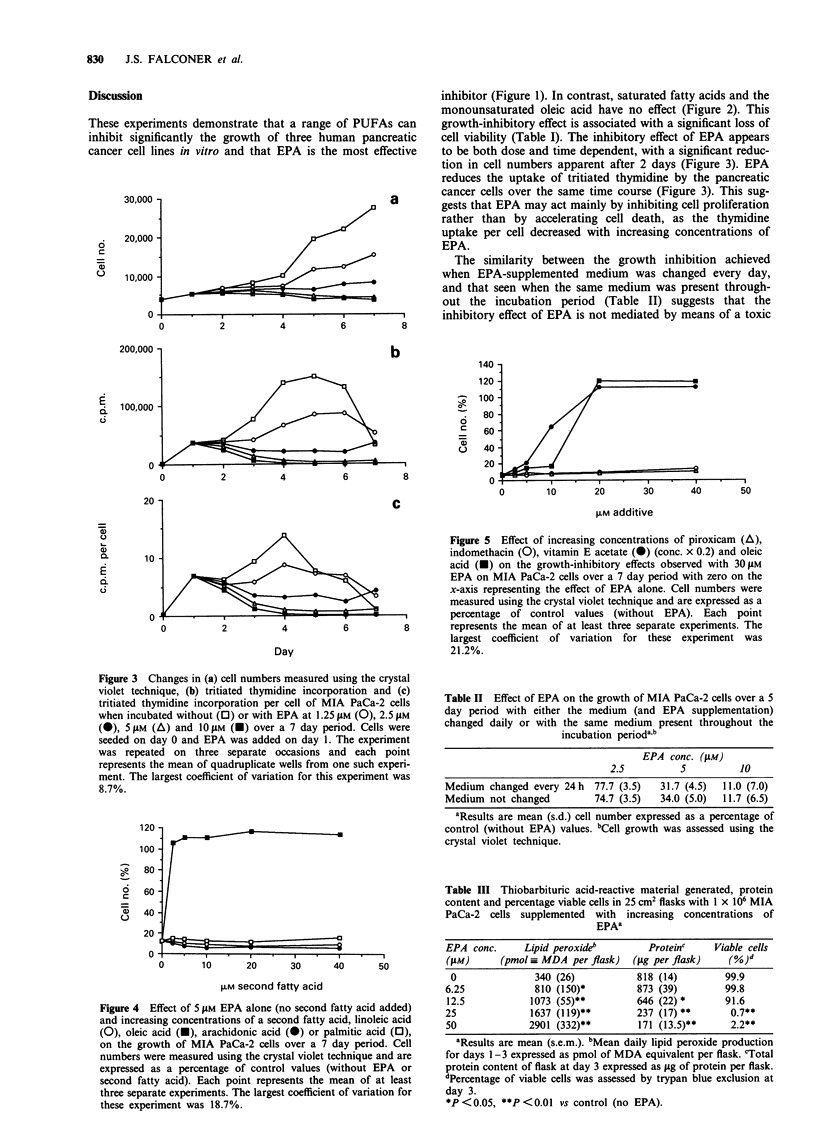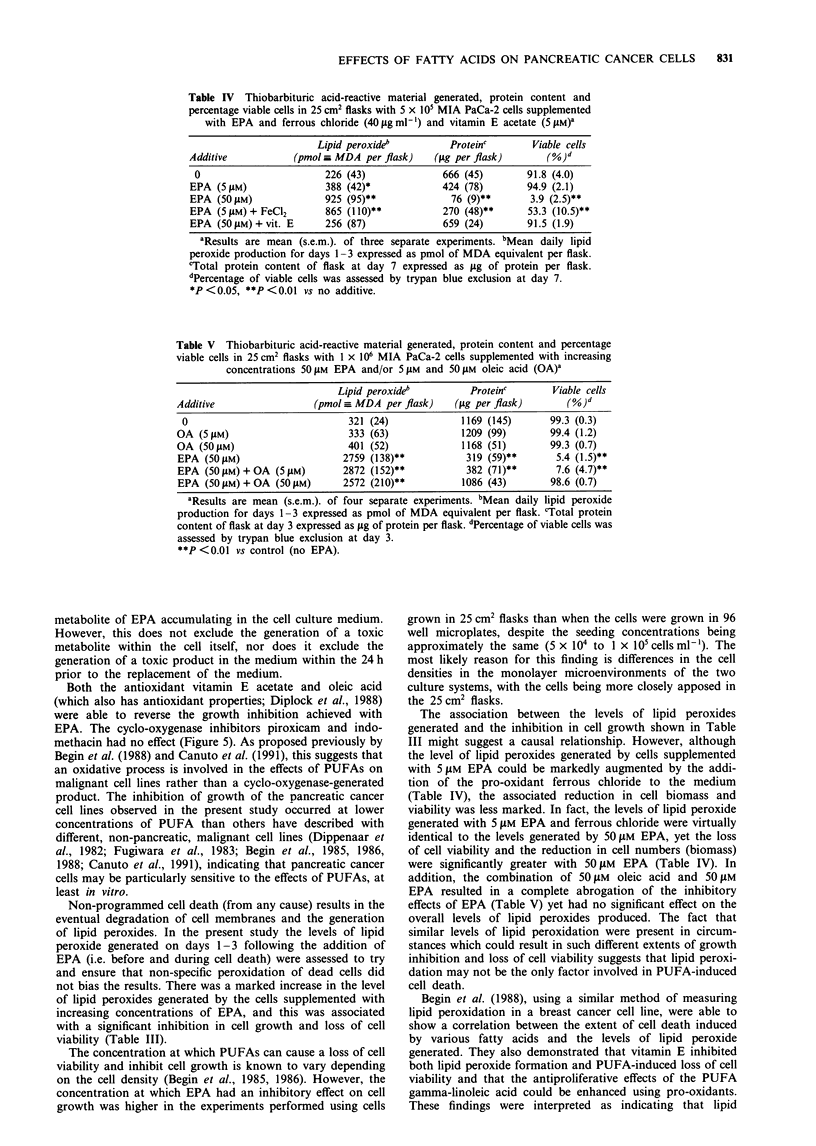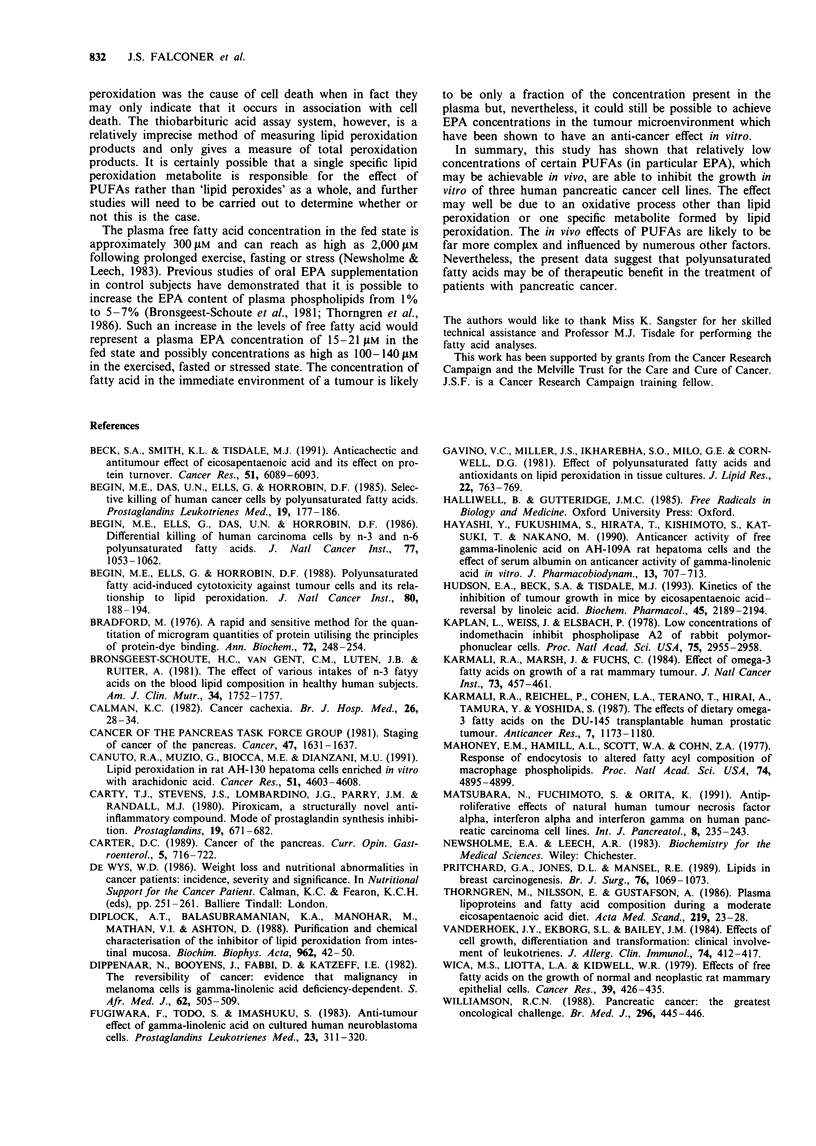# Effect of eicosapentaenoic acid and other fatty acids on the growth in vitro of human pancreatic cancer cell lines.

**DOI:** 10.1038/bjc.1994.161

**Published:** 1994-05

**Authors:** J. S. Falconer, J. A. Ross, K. C. Fearon, R. A. Hawkins, M. G. O'Riordain, D. C. Carter

**Affiliations:** University Department of Surgery, Royal Infirmary, Edinburgh, UK.

## Abstract

A number of polyunsaturated fatty acids have been shown to inhibit the growth of malignant cells in vitro. To investigate whether fatty acids modify the growth of human pancreatic cancer, lauric, stearic, palmitic, oleic, linoleic, alpha-linolenic, gamma-linolenic, arachidonic, docosahexaenoic and eicosapentaenoic (EPA) acids were each incubated with the cells lines MIA PaCa-2, PANC-1 and CFPAC at concentrations ranging from 1.25 microM to 50 microM and the effect of each fatty acid on cell growth was examined. All the polyunsaturated fatty acids tested had an inhibitory effect, with EPA being the most potent (ID50 2.5-5 microM). Monounsaturated or saturated fatty acids were not inhibitory. The action of EPA could be reversed with the anti-oxidant vitamin E acetate or with oleic acid. The cyclo-oxygenase inhibitors indomethacin and piroxicam had no effect on the action of EPA. The action of EPA appeared to be associated with the generation of lipid peroxides, although the level of lipid peroxidation did not always appear to correlate directly with the extent of cell death. The ability of certain fatty acids to inhibit significantly the growth of three human pancreatic cancer cell lines in vitro at concentrations which could be achieved in vivo suggests that administration of such fatty acids may be of therapeutic benefit in patients with pancreatic cancer.


Br. J. Cancer (1994), 69, 826-832 © Macmillan Press Ltd., 1994

### Effect of eicosapentaenoic acid and other fatty acids on the growth *in vitro* of human pancreatic cancer cell lines

J.S. Falconer, J.A. Ross, K.C.H. Fearon, R.A. Hawkins, M.G. O'Riordain & D.C. Carter

*University Department of Surgery, Royal Infirmary, Edinburgh, EH3 9YW, UK.*

**Summary** A number of polyunsaturated fatty acids have been shown to inhibit the growth of malignant cells *in vitro*. To investigate whether fatty acids modify the growth of human pancreatic cancer, lauric, stearic, palmitic, oleic, linoleic, alpha-linolenic, gamma-linolenic, arachidonic, docosahexaenoic and eicosapentaenoic (EPA) acids were each incubated with the cells lines MIA PaCa-2, PANC-1 and CFPAC at concentrations ranging from 1.25 μM to 50 μM and the effect of each fatty acid on cell growth was examined. All the polyunsaturated fatty acids tested had an inhibitory effect, with EPA being the most potent ($ID_{50}$ 2.5-5 μM). Monounsaturated or saturated fatty acids were not inhibitory. The action of EPA could be reversed with the anti-oxidant vitamin E acetate or with oleic acid. The cyclo-oxygenase inhibitors indomethacin and piroxicam had no effect on the action of EPA. The action of EPA appeared to be associated with the generation of lipid peroxides, although the level of lipid peroxidation did not always appear to correlate directly with the extent of cell death. The ability of certain fatty acids to inhibit significantly the growth of three human pancreatic cancer cell lines *in vitro* at concentrations which could be achieved *in vivo* suggests that administration of such fatty acids may be of therapeutic benefit in patients with pancreatic cancer.

Pancreatic cancer is now the fifth commonest cause of cancer death in the Western world (Williamson, 1988). Despite recent improvements in diagnosis and staging, the prognosis remains very poor, with a median survival of approximately 3-6 months (Cancer of the Pancreas Task Force Group, 1981; Williamson, 1988). Surgical resection of early disease offers the only chance of long-term survival but is rarely feasible since most patients present with advanced disease (Carter, 1989). Pancreatic cancer is also associated with substantial morbidity. For example, patients have a very high incidence of cachexia (DeWys, 1986), and indeed progressive weight loss is often the major symptom experienced. Clearly the best way to reverse such cachexia is to provide effective treatment of the cancer (Calman, 1982). However, at present there is no effective systemic antineoplastic therapy for advanced pancreatic cancer (Carter, 1989), and the toxicity of conventional chemotherapy may contribute further to the deteriorating nutritional status of the patient. Hence there is an urgent need for new, selective, non-toxic treatments for advanced pancreatic malignancy.

A number of studies have suggested that certain polyunsaturated fatty acids (PUFAs) can inhibit the growth of a variety of human cancer cell lines *in vitro* (Wica *et al.*, 1979; Dippenaar *et al.*, 1982; Fugiwara *et al.*, 1983; Begin *et al.*, 1985, 1986). Moreover, the effects of PUFAs have been shown to be selective for cancer cells without affecting normal cells *in vitro* (Begin *et al.*, 1986). Although enhanced lipid peroxidation has been proposed as one of the main mechanisms by which PUFAs inhibit tumour cell growth *in vitro* (Begin *et al.*, 1988), it is not clear whether this is the case with all cell lines, nor is it known which peroxidation products are important in tumour cell killing.

It has been suggested that the presence of albumin in the culture medium may decrease the anti-tumour effects of free fatty acids *in vitro* (Hayashi *et al.*, 1990), but this has not been tested using human cancer cell lines. In addition, a variety of antioxidants such as vitamin E, oleic acid and sodium selenite (Begin *et al.*, 1988) have been shown to decrease the anti-cancer effects of PUFAs *in vitro*. Since these antioxidants are often present *in vivo*, such findings bring into question whether PUFAs might be effective anticancer agents in patients. Recent studies have, however, demonstrated the growth-inhibitory effects of diets supplemented with the PUFAs eicosapentaenoic acid (EPA) and gamma-linolenic acid in a variety of tumour-bearing mouse models (Karmali *et al.*, 1984, 1987; Pritchard *et al.*, 1989; Beck *et al.*, 1991).

In order to test the potential of PUFAs for the treatment of patients with pancreatic cancer, the effects of a variety of fatty acids on the growth of three human pancreatic cancer cell lines was examined. Since free fatty acids are normally bound to albumin *in vivo*, fatty acids complexed to albumin were used. Lipid peroxidation and the metabolism of fatty acids by the cyclo-oxygenase pathway represent potential mechanisms for the inhibition of tumour cell growth by PUFAs. With a view to optimising the anti-cancer effects of PUFAs *in vivo*, the *in vitro* effects of manipulating these pathways were also studied.

### Materials and methods

### *Reagents*

Palmitic acid, stearic acid, lauric acid, linoleic acid, alpha-linolenic acid, arachidonic acid, gamma-linolenic acid, docosahexaenoic acid, eicosapentaenoic acid (EPA), fatty acid-free bovine serum albumin (BSA), indomethacin, vitamin E acetate, piroxicam and ferrous chloride were obtained from Sigma (Sigma, Poole, Dorset, UK). Malondialdehyde (MDA), thiobarbituric acid and trichloroacetic acid were obtained from BDH (Glasgow, UK). [$^3$H]thymidine was obtained from Amersham (Buckinghamshire, UK). Fatty acids were complexed to BSA according to the method of Mahoney *et al.* (1977) and 1 mM stock solutions were stored at −20°C. Subsequent analysis of the stock solutions by gas-liquid chromatography (Hudson *et al.*, 1993) indicated that there was no significant degradation of the fatty acids during the complexing process. Eicosanoid inhibitors were dissolved in 100% ethanol and ferrous chloride was dissolved in 150 mM sodium chloride and stock solutions were stored at −20°C.

### *Cell lines*

The human pancreatic cancer cell lines MIA PaCa-2, PANC-1 and CFPAC were obtained from the European Tissue Culture Collection, Porton Down, UK. Cell lines were routinely grown in Dulbecco's modified Eagle medium sup-

Correspondence: J.A. Ross.
Received 3 September 1993; and in revised form 17 November 1993.

plemented with 5% fetal bovine serum (ICN Biomedicals, Irvine, Ayrshire, UK), 1 mM glutamine and penicillin/streptomycin (Sigma) in a 95% air/5% carbon dioxide humidified incubator. The same batch of fetal bovine serum was used for all experiments to minimise effects due to inter-batch variability.

*Growth experiments*

Cells were routinely seeded in 96 well flat-bottomed microplates (Costar Corporation, Cambridge, UK) at a density of $5 \times 10^3$ cells per well in 100 µl of medium and incubated for 24 h before supplementation of the medium with fatty acid complexed to BSA. Fatty acid and/or eicosanoid inhibitors were added to give a final volume of 120 µl. When agents dissolved in ethanol were added the final concentration of ethanol was 0.5% or less and ethanol alone was added to the control wells. In experiments designed to examine the effect of fatty acid alone, cells were routinely incubated for a further 6 days with the relevant fatty acid. At the end of the incubation period cell numbers were assessed by a modification of the method of Matsubara *et al.* (1991). Briefly, the medium was removed and any adherent cells were fixed to the plate with 5% formaldehyde in phosphate-buffered saline (PBS). The cells were then stained with a 0.5% aqueous solution of crystal violet followed by elution of the dye with 33% aqueous acetic acid. Absorbance at 570 nm was determined with a Dynatech 5000 microplate reader (Dynatech Laboratories, Billingshurst, West Sussex, UK) and the number of cells was determined from a standard curve of absorbance against cell numbers calculated from a mean of six experiments for each cell line ($R^2 = 0.99$). When the effect of fatty acids on cell viability was assessed, this was measured by trypan blue exclusion and the results were expressed as the percentage of viable cells at the end of the incubation period. When thymidine uptake was measured, the cells were incubated with 1 µCi of [$^3$H]thymidine for 4 h at the end of the incubation period. The cells were then harvested onto filter paper using a Dynatech AUTOMASH 2000 cell harvester, placed in 5 ml of liquid scintillant and [$^3$H]thymidine uptake was measured using a Packard Tri-carb 300C counter (Packard Instrument, Downers Grove, IL, USA).

*Lipid peroxidation experiments*

Polyunsaturated lipids are highly susceptible to lipid peroxidation, giving rise to various alkenal and aldehyde metabolites (Halliwell & Gutteridge, 1985). A number of these metabolites, including malondialdehyde (MDA), react with thiobarbituric acid to produce a pink-coloured material that can readily be monitored by spectrophotometry to give an overall indication of the level of lipid peroxidation (Halliwell & Gutteridge, 1985). Cells were cultured in 25 cm$^2$ flasks at an initial concentration of $5 \times 10^5$ or $1 \times 10^6$ cells in 10 ml of standard medium. EPA supplementation and other additions were carried out after 24 h incubation. Lipid peroxidation was measured according to the method of Gavino *et al.* (1981). Briefly, at the end of the incubation period, the supernatant was removed and centrifuged to recover any non-adherent cells. Both non-adherent cells and cells adherent to the flask were washed with 0.9% sodium chloride until all the colour of the medium was removed and then the pooled cells were resuspended in a final volume of 2 ml of PBS. Two millilitres of 20% trichloroacetic acid and 2 ml of 0.67% thiobarbituric acid was then added, this mixture was incubated at 90°C for 20 min and the supernatant was then centrifuged to remove any debris. The absorbance of the supernatant at 532 nm was then measured against that of a reagent blank treated similarly but containing no cells. Absorbance was converted to picomoles (pmol) of MDA equivalents using a standard curve generated with MDA, 62.5- 6,250 pmol ($R^2 = 1.00$ in three experiments).

In addition, the total protein content of the flasks was assayed to give a measure of the biomass of cells in the flasks. Following the measurement of lipid peroxides, the cells were removed from the flasks, resuspended in 0.9% sodium chloride and washed twice with 0.9% sodium chloride. A 450 µl volume of 2 M sodium hydroxide was then added to the cell pellet, which was left for 18 h at room temperature followed by 1 h of heating at 60°C to dissolve the pellet. The resulting solution was partially neutralised with 350 µl of 2 M hydrochloric acid and aliquots were then assayed for protein content using the Coomassie brilliant blue method of Bradford (1976) with BSA as a standard. Biomass was expressed as micrograms of protein per flask. A duplicate set of flasks were processed to allow the assessment of cell viability by the method of trypan blue exclusion. Results were expressed as the percentage of viable cells at the end of the incubation period.

*Statistics*

Statistical analysis was carried out using a two-tailed Student's *t*-test, and differences were regarded as significant when the chance of their arising by sampling error was less than 1 in 20 ($P < 0.05$).

### Results

*Effects of fatty acids on cell numbers*

All of the PUFAs tested (alpha-linolenic acid, linoleic acid, arachidonic acid, docosahexaenoic acid, gamma-linolenic acid and EPA) significantly reduced the growth rate of the three pancreatic cancer cell lines in a dose-dependent manner (Figure 1). Of the PUFAs tested, EPA was the most effective inhibitor, with a greater than 90% reduction in cell numbers at the highest concentrations tested.

In contrast, none of the saturated fatty acids (palmitic acid, stearic acid and lauric acid) or the monounsaturated acid (oleic acid) had any inhibitory effect on growth (Figure 2). Indeed, palmitic acid, lauric acid and oleic acid all significantly enhanced growth at some concentrations (Figure 2).

*Effects of fatty acids on cell viability*

The effect on the viability of MIA PaCa-2 cells after 6 days' incubation of EPA, gamma-linolenic acid, oleic acid and palmitic acid at a variety of concentrations is shown in Table I. Both PUFAs resulted in a significant loss of viability in a dose-dependent manner, with EPA being the more effective. Oleic acid and palmitic acid had no significant effect.

*Effects of EPA on cell proliferation*

In order to discriminate between an effect of EPA on the rate of cell growth as opposed to cell death, thymidine uptake was used as an index of DNA synthesis, while cell number was used to estimate any net change in biomass. Figure 3 shows the effect of increasing concentrations of EPA on the growth of MIA PaCa-2 over a 7 day period when both absolute cell number and tritiated thymidine uptake were measured. A significant inhibitory effect of EPA on cell numbers was observed after 3 days' incubation, and the magnitude of this inhibition increased with time. Similarly, the inhibition of thymidine uptake by EPA became apparent after 2–3 days and the magnitude of the inhibition increased with time. Expression of thymidine uptake per cell also revealed that the inhibitory effect of EPA only became apparent after 3 days' incubation and that the magnitude of the effect increased with increasing concentrations of EPA.

*Effect of medium replenishment*

In order to establish whether the effect of EPA may be mediated indirectly via a metabolic or degradation product of EPA accumulating in the culture medium, MIA PaCa-2

**Figure 1** Effect of increasing concentrations of the fatty acids (**a**) alpha-linolenic acid (ALA), (**b**) linoleic acid (LA), (**c**) arachidonic acid (AA), (**d**) gamma-linolenic acid (GLA), (**e**) docosahexaenoic acid (DHA) and (**f**) eicosapentaenoic acid (EPA) on the growth of the three human pancreatic cancer cell lines MIA PaCa-2 (□), PANC-1 (○) and CFPAC (■) over a 7 day period. Cell numbers were measured using the crystal violet technique and are expressed as a percentage of control values (without fatty acid). Each point represents the mean of at least three separate experiments. The largest coefficient of variation for each set of experiments was (**a**) 20.0%, (**b**) 18.4%, (**c**) 7.9%, (**d**) 8.4%, (**e**) 11.6% and (**f**) 6.4%.

cells were incubated with and without EPA supplementation and were either left to grow in the medium over a 5 day period or had the medium and EPA supplementation changed daily. The results are shown in Table II. There was no significant difference between the inhibition achieved with EPA at various concentrations whether or not the EPA-supplemented medium was replaced on a daily basis.

*Effects of various fatty acids in combination*

Figure 4 shows the effect of EPA alone and in combination with the fatty acids oleic acid, linoleic acid, arachidonic acid and palmitic acid at increasing concentrations. Oleic acid at 2.5 μM abrogated the inhibitory effect of EPA completely and, of the fatty acids tested, this effect was unique to oleic acid.

*Effects of antioxidants and inhibitors*

The effects of a variety of antioxidants and inhibitors at increasing concentrations in combination with a fixed concentration of 30 μM EPA on MIA PaCa-2 cells are shown in Figure 5. The agents examined were oleic acid, vitamin E acetate (antioxidant), piroxicam (cyclo-oxygenase inhibitor; Carty et al., 1980) and indomethacin [which is primarily a cyclo-oxygenase inhibitor but at high concentrations is able to inhibit both phospholipase $A_2$ (Kaplan et al., 1978) and lipoxygenase (Vanderhoek et al., 1984)]. Only oleic acid and the antioxidant vitamin E acetate were able to abrogate the effect of EPA, with both piroxicam and indomethacin having no effect.

*The role of lipid peroxidation*

The relationship between the generation of lipid peroxides (measured as thiobarbituric acid-reactive material), cell growth (measured as the protein content of the culture flasks) and cell viability in flasks seeded with $1 \times 10^6$ MIA PaCa-2 cells supplemented with increasing concentrations of EPA is shown in Table III. As the concentration of EPA increased, so the level of lipid peroxides increased, and this was associated with loss of cell viability.

The effects of EPA, vitamin E acetate and ferrous chloride on lipid peroxide production, cell growth and cell viability

**Figure 2** Effect of increasing concentrations of the fatty acids (**a**) stearic acid (SA), (**b**) lauric acid (Lau A), (**c**) palmitic acid (PA) and (**d**) oleic acid (OA) on the growth of the three human pancreatic cancer cell lines MIA PaCa-2 (□), PANC-1 (○) and CFPAC (■) over a 7 day period. Cell numbers were measured using the crystal violet technique and are expressed as a percentage of control values (without fatty acid). Each point represents the mean of at least three separate experiments. The largest coefficient of variation for each set of experiments was (**a**) 13.4%, (**b**) 21.3%, (**c**) 19.6% and (**d**) 12.4%.

**Table I** Effect on viability of MIA PaCa-2 cells after 6 days' incubation with different concentrations of EPA, gamma-linolenic acid (GLA), oleic acid (OA) and palmitic acid (PA)[a,b]

| *Fatty acid conc.* | *Viable cells (%)* | | | |
|---|---|---|---|---|
| *(μM)* | *EPA* | *GLA* | *OA* | *PA* |
| 0 | 94.7 (3.4) | 96.2 (3.7) | 98.7 (2.9) | 93.7 (3.5) |
| 2.5 | 74.7 (12.4)* | 89.1 (7.9) | 97.3 (2.2) | 92.5 (6.9) |
| 5 | 56.0 (4.1)** | 68.4 (6.7)* | 96.8 (2.6) | 97.5 (4.8) |
| 10 | 33.3 (4.0)** | 41.3 (4.6)** | 98.0 (5.8) | 93.8 (2.5) |
| 20 | 8.5 (2.5)** | 27.0 (3.7)** | 95.0 (2.9) | 94.2 (2.5) |
| 40 | 5.7 (3.4)** | 11.3 (5.7)** | 98.8 (1.7) | 98.0 (2.0) |

[a]Results are mean (s.d.). [b]Cell viability was assessed using trypan blue exclusion. $^{*}P < 0.05$, $^{**}P < 0.01$ *vs* control (no fatty acid supplementation).

using flasks seeded with $5 \times 10^5$ MIA PaCa-2 cells is shown in Table IV. At low concentrations of EPA (5 μM), cell growth and viability were not inhibited significantly. There was, however, a small but significant increase in the level of thiobarbituric acid-reactive material produced compared with that found in cells cultured in the absence of EPA. At higher concentrations of EPA (50 μM), cell protein mass was reduced significantly, as was cell viability. This was associated with a significant increase in the levels of thiobarbituric acid-reactive material when compared with cells not supplemented with EPA. The pro-oxidant ferrous chloride significantly enhanced both the levels of lipid peroxide and the inhibitory effect of EPA (5 μM) on cell growth and cell viability. Conversely, in the presence of the antioxidant vitamin E acetate, the effect of EPA (50 μM) on cell growth and the level of lipid peroxidation products generated was completely reversed.

Table V shows the results of a similar set of experiments using flasks seeded with $1 \times 10^6$ MIA PaCa-2 cells where EPA was added in combination with oleic acid. Oleic acid had been shown to abrogate the growth-inhibitory effects of EPA in the 96 well microplate experimental system (Figures 4 and 5). In the experiments shown in Table V, the greater cell density resulted in the levels of peroxide generated being greater than in the previous set of experiments (see Table IV). Again 50 μM EPA caused a significant reduction in cell growth and cell viability, and this was associated with increased generation of lipid peroxides. Oleic acid alone at concentrations of 5 μM and 50 μM had no effect on cell growth or peroxide formation. Supplementation of medium with 50 μM and 5 μM oleic acid did not alter the effects observed with 50 μM EPA alone. In contrast, the combination of 50 μM oleic acid with 50 μM EPA resulted in a complete abrogation of the growth-inhibitory effects of EPA, but the total levels of lipid peroxides generated were unaltered and were not significantly different from those seen with EPA alone, although the amount of lipid peroxides generated on day 1 was lower in the flasks containing 50 μM oleic acid and 50 μM EPA.

### Discussion

These experiments demonstrate that a range of PUFAs can inhibit significantly the growth of three human pancreatic cancer cell lines *in vitro* and that EPA is the most effective inhibitor (Figure 1). In contrast, saturated fatty acids and the monounsaturated oleic acid have no effect (Figure 2). This growth-inhibitory effect is associated with a significant loss of cell viability (Table I). The inhibitory effect of EPA appears to be both dose and time dependent, with a significant reduction in cell numbers apparent after 2 days (Figure 3). EPA reduces the uptake of tritiated thymidine by the pancreatic cancer cells over the same time course (Figure 3). This suggests that EPA may act mainly by inhibiting cell proliferation rather than by accelerating cell death, as the thymidine uptake per cell decreased with increasing concentrations of EPA.

The similarity between the growth inhibition achieved when EPA-supplemented medium was changed every day, and that seen when the same medium was present throughout the incubation period (Table II) suggests that the inhibitory effect of EPA is not mediated by means of a toxic

**Figure 3** Changes in (**a**) cell numbers measured using the crystal violet technique, (**b**) tritiated thymidine incorporation and (**c**) tritiated thymidine incorporation per cell of MIA PaCa-2 cells when incubated without (□) or with EPA at 1.25 µM (○), 2.5 µM (●), 5 µM (△) and 10 µM (■) over a 7 day period. Cells were seeded on day 0 and EPA was added on day 1. The experiment was repeated on three separate occasions and each point represents the mean of quadruplicate wells from one such experiment. The largest coefficient of variation for this experiment was 8.7%.

**Figure 4** Effect of 5 µM EPA alone (no second fatty acid added) and increasing concentrations of a second fatty acid, linoleic acid (○), oleic acid (■), arachidonic acid (●) or palmitic acid (□), on the growth of MIA PaCa-2 cells over a 7 day period. Cell numbers were measured using the crystal violet technique and are expressed as a percentage of control values (without EPA or second fatty acid). Each point represents the mean of at least three separate experiments. The largest coefficient of variation for these experiment was 18.7%.

**Figure 5** Effect of increasing concentrations of piroxicam (△), indomethacin (○), vitamin E acetate (●) (conc. × 0.2) and oleic acid (■) on the growth-inhibitory effects observed with 30 µM EPA on MIA PaCa-2 cells over a 7 day period with zero on the *x*-axis representing the effect of EPA alone. Cell numbers were measured using the crystal violet technique and are expressed as a percentage of control values (without EPA). Each point represents the mean of at least three separate experiments. The largest coefficient of variation for these experiment was 21.2%.

**Table II** Effect of EPA on the growth of MIA PaCa-2 cells over a 5 day period with either the medium (and EPA supplementation) changed daily or with the same medium present throughout the incubation period[a,b]

| | *EPA conc. (µM)* | | |
|---|---|---|---|
| | *2.5* | *5* | *10* |
| Medium changed every 24 h | 77.7 (3.5) | 31.7 (4.5) | 11.0 (7.0) |
| Medium not changed | 74.7 (3.5) | 34.0 (5.0) | 11.7 (6.5) |

[a]Results are mean (s.d.) cell number expressed as a percentage of control (without EPA) values. [b]Cell growth was assessed using the crystal violet technique.

**Table III** Thiobarbituric acid-reactive material generated, protein content and percentage viable cells in 25 cm$^2$ flasks with 1 × 10$^6$ MIA PaCa-2 cells supplemented with increasing concentrations of EPA[a]

| *EPA conc. (µM)* | *Lipid peroxide[b] (pmol ≡ MDA per flask)* | *Protein[c] (µg per flask)* | *Viable cells (%)[d]* |
|---|---|---|---|
| 0 | 340 (26) | 818 (14) | 99.9 |
| 6.25 | 810 (150)* | 873 (39) | 99.8 |
| 12.5 | 1073 (55)** | 646 (22) * | 91.6 |
| 25 | 1637 (119)** | 237 (17) ** | 0.7** |
| 50 | 2901 (332)** | 171 (13.5)** | 2.2** |

[a]Results are mean (s.e.m.). [b]Mean daily lipid peroxide production for days 1–3 expressed as pmol of MDA equivalent per flask. [c]Total protein content of flask at day 3 expressed as µg of protein per flask. [d]Percentage of viable cells was assessed by trypan blue exclusion at day 3.
*$P$ < 0.05, **$P$ < 0.01 *vs* control (no EPA).

**Table IV** Thiobarbituric acid-reactive material generated, protein content and percentage viable cells in 25 cm$^2$ flasks with 5 × 10$^5$ MIA PaCa-2 cells supplemented with EPA and ferrous chloride (40 μg ml$^{-1}$) and vitamin E acetate (5 μM)[a]

| *Additive* | *Lipid peroxide*[b] *(pmol ≡ MDA per flask)* | *Protein*[c] *(μg per flask)* | *Viable cells (%)*[d] |
|---|---|---|---|
| 0 | 226 (43) | 666 (45) | 91.8 (4.0) |
| EPA (5 μM) | 388 (42)* | 424 (78) | 94.9 (2.1) |
| EPA (50 μM) | 925 (95)** | 76 (9)** | 3.9 (2.5)** |
| EPA (5 μM) + $FeCl_2$ | 865 (110)** | 270 (48)** | 53.3 (10.5)** |
| EPA (50 μM) + vit. E | 256 (87) | 659 (24) | 91.5 (1.9) |

[a]Results are mean (s.e.m.). of three separate experiments. [b]Mean daily lipid peroxide production for days 1–3 expressed as pmol of MDA equivalent per flask. [c]Total protein content of flask at day 7 expressed as μg of protein per flask. [d]Percentage of viable cells was assessed by trypan blue exclusion at day 7.
*$P < 0.05$, **$P < 0.01$ *vs* no additive.

**Table V** Thiobarbituric acid-reactive material generated, protein content and percentage viable cells in 25 cm$^2$ flasks with 1 × 10$^6$ MIA PaCa-2 cells supplemented with increasing concentrations 50 μM EPA and/or 5 μM and 50 μM oleic acid (OA)[a]

| *Additive* | *Lipid peroxide*[b] *(pmol ≡ MDA per flask)* | *Protein*[c] *(μg per flask)* | *Viable cells (%)*[d] |
|---|---|---|---|
| 0 | 321 (24) | 1169 (145) | 99.3 (0.3) |
| OA (5 μM) | 333 (63) | 1209 (99) | 99.4 (1.2) |
| OA (50 μM) | 401 (52) | 1168 (51) | 99.3 (0.7) |
| EPA (50 μM) | 2759 (138)** | 319 (59)** | 5.4 (1.5)** |
| EPA (50 μM) + OA (5 μM) | 2872 (152)** | 382 (71)** | 7.6 (4.7)** |
| EPA (50 μM) + OA (50 μM) | 2572 (210)** | 1086 (43) | 98.6 (0.7) |

[a]Results are mean (s.e.m.). of four separate experiments. [b]Mean daily lipid peroxide production for days 1–3 expressed as pmol of MDA equivalent per flask. [c]Total protein content of flask at day 3 expressed as μg of protein per flask. [d]Percentage of viable cells was assessed by trypan blue exclusion at day 3.
**$P < 0.01$ *vs* control (no EPA).

metabolite of EPA accumulating in the cell culture medium. However, this does not exclude the generation of a toxic metabolite within the cell itself, nor does it exclude the generation of a toxic product in the medium within the 24 h prior to the replacement of the medium.

Both the antioxidant vitamin E acetate and oleic acid (which also has antioxidant properties; Diplock *et al.*, 1988) were able to reverse the growth inhibition achieved with EPA. The cyclo-oxygenase inhibitors piroxicam and indomethacin had no effect (Figure 5). As proposed previously by Begin *et al.* (1988) and Canuto *et al.* (1991), this suggests that an oxidative process is involved in the effects of PUFAs on malignant cell lines rather than a cyclo-oxygenase-generated product. The inhibition of growth of the pancreatic cancer cell lines observed in the present study occurred at lower concentrations of PUFA than others have described with different, non-pancreatic, malignant cell lines (Dippenaar *et al.*, 1982; Fugiwara *et al.*, 1983; Begin *et al.*, 1985, 1986, 1988; Canuto *et al.*, 1991), indicating that pancreatic cancer cells may be particularly sensitive to the effects of PUFAs, at least *in vitro*.

Non-programmed cell death (from any cause) results in the eventual degradation of cell membranes and the generation of lipid peroxides. In the present study the levels of lipid peroxide generated on days 1–3 following the addition of EPA (i.e. before and during cell death) were assessed to try and ensure that non-specific peroxidation of dead cells did not bias the results. There was a marked increase in the level of lipid peroxides generated by the cells supplemented with increasing concentrations of EPA, and this was associated with a significant inhibition in cell growth and loss of cell viability (Table III).

The concentration at which PUFAs can cause a loss of cell viability and inhibit cell growth is known to vary depending on the cell density (Begin *et al.*, 1985, 1986). However, the concentration at which EPA had an inhibitory effect on cell growth was higher in the experiments performed using cells grown in 25 cm$^2$ flasks than when the cells were grown in 96 well microplates, despite the seeding concentrations being approximately the same (5 × 10$^4$ to 1 × 10$^5$ cells ml$^{-1}$). The most likely reason for this finding is differences in the cell densities in the monolayer microenvironments of the two culture systems, with the cells being more closely apposed in the 25 cm$^2$ flasks.

The association between the levels of lipid peroxides generated and the inhibition in cell growth shown in Table III might suggest a causal relationship. However, although the level of lipid peroxides generated by cells supplemented with 5 μM EPA could be markedly augmented by the addition of the pro-oxidant ferrous chloride to the medium (Table IV), the associated reduction in cell biomass and viability was less marked. In fact, the levels of lipid peroxide generated with 5 μM EPA and ferrous chloride were virtually identical to the levels generated by 50 μM EPA, yet the loss of cell viability and the reduction in cell numbers (biomass) were significantly greater with 50 μM EPA (Table IV). In addition, the combination of 50 μM oleic acid and 50 μM EPA resulted in a complete abrogation of the inhibitory effects of EPA (Table V) yet had no significant effect on the overall levels of lipid peroxides produced. The fact that similar levels of lipid peroxidation were present in circumstances which could result in such different extents of growth inhibition and loss of cell viability suggests that lipid peroxidation may not be the only factor involved in PUFA-induced cell death.

Begin *et al.* (1988), using a similar method of measuring lipid peroxidation in a breast cancer cell line, were able to show a correlation between the extent of cell death induced by various fatty acids and the levels of lipid peroxide generated. They also demonstrated that vitamin E inhibited both lipid peroxide formation and PUFA-induced loss of cell viability and that the antiproliferative effects of the PUFA gamma-linoleic acid could be enhanced using pro-oxidants. These findings were interpreted as indicating that lipid peroxidation was the cause of cell death when in fact they may only indicate that it occurs in association with cell death. The thiobarbituric acid assay system, however, is a relatively imprecise method of measuring lipid peroxidation products and only gives a measure of total peroxidation products. It is certainly possible that a single specific lipid peroxidation metabolite is responsible for the effect of PUFAs rather than 'lipid peroxides' as a whole, and further studies will need to be carried out to determine whether or not this is the case.

The plasma free fatty acid concentration in the fed state is approximately 300 μM and can reach as high as 2,000 μM following prolonged exercise, fasting or stress (Newsholme & Leech, 1983). Previous studies of oral EPA supplementation in control subjects have demonstrated that it is possible to increase the EPA content of plasma phospholipids from 1% to 5–7% (Bronsgeest-Schoute *et al.*, 1981; Thorngren *et al.*, 1986). Such an increase in the levels of free fatty acid would represent a plasma EPA concentration of 15–21 μM in the fed state and possibly concentrations as high as 100–140 μM in the exercised, fasted or stressed state. The concentration of fatty acid in the immediate environment of a tumour is likely to be only a fraction of the concentration present in the plasma but, nevertheless, it could still be possible to achieve EPA concentrations in the tumour microenvironment which have been shown to have an anti-cancer effect *in vitro*.

In summary, this study has shown that relatively low concentrations of certain PUFAs (in particular EPA), which may be achievable *in vivo*, are able to inhibit the growth *in vitro* of three human pancreatic cancer cell lines. The effect may well be due to an oxidative process other than lipid peroxidation or one specific metabolite formed by lipid peroxidation. The *in vivo* effects of PUFAs are likely to be far more complex and influenced by numerous other factors. Nevertheless, the present data suggest that polyunsaturated fatty acids may be of therapeutic benefit in the treatment of patients with pancreatic cancer.

The authors would like to thank Miss K. Sangster for her skilled technical assistance and Professor M.J. Tisdale for performing the fatty acid analyses.

This work has been supported by grants from the Cancer Research Campaign and the Melville Trust for the Care and Cure of Cancer. J.S.F. is a Cancer Research Campaign training fellow.

### References

BECK, S.A., SMITH, K.L. & TISDALE, M.J. (1991). Anticachectic and antitumour effect of eicosapentaenoic acid and its effect on protein turnover. *Cancer Res.*, **51**, 6089–6093.

BEGIN, M.E., DAS, U.N., ELLS, G. & HORROBIN, D.F. (1985). Selective killing of human cancer cells by polyunsaturated fatty acids. *Prostaglandins Leukotrienes Med.*, **19**, 177–186.

BEGIN, M.E., ELLS, G., DAS, U.N. & HORROBIN, D.F. (1986). Differential killing of human carcinoma cells by n-3 and n-6 polyunsaturated fatty acids. *J. Natl Cancer Inst.*, **77**, 1053–1062.

BEGIN, M.E., ELLS, G. & HORROBIN, D.F. (1988). Polyunsaturated fatty acid-induced cytotoxicity against tumour cells and its relationship to lipid peroxidation. *J. Natl Cancer Inst.*, **80**, 188–194.

BRADFORD, M. (1976). A rapid and sensitive method for the quantitation of microgram quantities of protein utilising the principles of protein-dye binding. *Ann. Biochem.*, **72**, 248–254.

BRONSGEEST-SCHOUTE, H.C., VAN GENT, C.M., LUTEN, J.B. & RUITER, A. (1981). The effect of various intakes of n-3 fatyy acids on the blood lipid composition in healthy human subjects. *Am. J. Clin. Mutr.*, **34**, 1752–1757.

CALMAN, K.C. (1982). Cancer cachexia. *Br. J. Hosp. Med.*, **26**, 28–34.

CANCER OF THE PANCREAS TASK FORCE GROUP (1981). Staging of cancer of the pancreas. *Cancer*, **47**, 1631–1637.

CANUTO, R.A., MUZIO, G., BIOCCA, M.E. & DIANZANI, M.U. (1991). Lipid peroxidation in rat AH-130 hepatoma cells enriched *in vitro* with arachidonic acid. *Cancer Res.*, **51**, 4603–4608.

CARTY, T.J., STEVENS, J.S., LOMBARDINO, J.G., PARRY, J.M. & RANDALL, M.J. (1980). Piroxicam, a structurally novel anti-inflammatory compound. Mode of prostaglandin synthesis inhibition. *Prostaglandins*, **19**, 671–682.

CARTER, D.C. (1989). Cancer of the pancreas. *Curr. Opin. Gastroenterol.*, **5**, 716–722.

DE WYS, W.D. (1986). Weight loss and nutritional abnormalities in cancer patients: incidence, severity and significance. In *Nutritional Support for the Cancer Patient*. Calman, K.C. & Fearon, K.C.H. (eds), pp. 251–261. Balliere Tindall: London.

DIPLOCK, A.T., BALASUBRAMANIAN, K.A., MANOHAR, M., MATHAN, V.I. & ASHTON, D. (1988). Purification and chemical characterisation of the inhibitor of lipid peroxidation from intestinal mucosa. *Biochim. Biophys. Acta*, **962**, 42–50.

DIPPENAAR, N., BOOYENS, J., FABBI, D. & KATZEFF, I.E. (1982). The reversibility of cancer: evidence that malignancy in melanoma cells is gamma-linolenic acid deficiency-dependent. *S. Afr. Med. J.*, **62**, 505–509.

FUGIWARA, F., TODO, S. & IMASHUKU, S. (1983). Anti-tumour effect of gamma-linolenic acid on cultured human neuroblastoma cells. *Prostaglandins Leukotrienes Med.*, **23**, 311–320.

GAVINO, V.C., MILLER, J.S., IKHAREBHA, S.O., MILO, G.E. & CORNWELL, D.G. (1981). Effect of polyunsaturated fatty acids and antioxidants on lipid peroxidation in tissue cultures. *J. Lipid Res.*, **22**, 763–769.

HALLIWELL, B. & GUTTERIDGE, J.M.C. (1985). *Free Radicals in Biology and Medicine*. Oxford University Press: Oxford.

HAYASHI, Y., FUKUSHIMA, S., HIRATA, T., KISHIMOTO, S., KATSUKI, T. & NAKANO, M. (1990). Anticancer activity of free gamma-linolenic acid on AH-109A rat hepatoma cells and the effect of serum albumin on anticancer activity of gamma-linolenic acid *in vitro*. *J. Pharmacobiodynam.*, **13**, 707–713.

HUDSON, E.A., BECK, S.A. & TISDALE, M.J. (1993). Kinetics of the inhibition of tumour growth in mice by eicosapentaenoic acid–reversal by linoleic acid. *Biochem. Pharmacol.*, **45**, 2189–2194.

KAPLAN, L., WEISS, J. & ELSBACH, P. (1978). Low concentrations of indomethacin inhibit phospholipase A2 of rabbit polymorphonuclear cells. *Proc. Natl Acad. Sci. USA*, **75**, 2955–2958.

KARMALI, R.A., MARSH, J. & FUCHS, C. (1984). Effect of omega-3 fatty acids on growth of a rat mammary tumour. *J. Natl Cancer Inst.*, **73**, 457–461.

KARMALI, R.A., REICHEL, P., COHEN, L.A., TERANO, T., HIRAI, A., TAMURA, Y. & YOSHIDA, S. (1987). The effects of dietary omega-3 fatty acids on the DU-145 transplantable human prostatic tumour. *Anticancer Res.*, **7**, 1173–1180.

MAHONEY, E.M., HAMILL, A.L., SCOTT, W.A. & COHN, Z.A. (1977). Response of endocytosis to altered fatty acyl composition of macrophage phospholipids. *Proc. Natl Acad. Sci. USA*, **74**, 4895–4899.

MATSUBARA, N., FUCHIMOTO, S. & ORITA, K. (1991). Antiproliferative effects of natural human tumour necrosis factor alpha, interferon alpha and interferon gamma on human pancreatic carcinoma cell lines. *Int. J. Pancreatol.*, **8**, 235–243.

NEWSHOLME, E.A. & LEECH, A.R. (1983). *Biochemistry for the Medical Sciences*. Wiley: Chichester.

PRITCHARD, G.A., JONES, D.L. & MANSEL, R.E. (1989). Lipids in breast carcinogenesis. *Br. J. Surg.*, **76**, 1069–1073.

THORNGREN, M., NILSSON, E. & GUSTAFSON, A. (1986). Plasma lipoproteins and fatty acid composition during a moderate eicosapentaenoic acid diet. *Acta Med. Scand.*, **219**, 23–28.

VANDERHOEK, J.Y., EKBORG, S.L. & BAILEY, J.M. (1984). Effects of cell growth, differentiation and transformation: clinical involvement of leukotrienes. *J. Allerg. Clin. Immunol.*, **74**, 412–417.

WICA, M.S., LIOTTA, L.A. & KIDWELL, W.R. (1979). Effects of free fatty acids on the growth of normal and neoplastic rat mammary epithelial cells. *Cancer Res.*, **39**, 426–435.

WILLIAMSON, R.C.N. (1988). Pancreatic cancer: the greatest oncological challenge. *Br. Med. J.*, **296**, 445–446.